# Vascular Calcification and Renal Bone Disorders

**DOI:** 10.1155/2014/637065

**Published:** 2014-07-17

**Authors:** Kuo-Cheng Lu, Chia-Chao Wu, Jen-Fen Yen, Wen-Chih Liu

**Affiliations:** ^1^Division of Nephrology, Department of Medicine, Cardinal Tien Hospital, School of Medicine, Fu Jen Catholic University, New Taipei City 23148, Taiwan; ^2^Division of Nephrology, Department of Medicine, Tri-Service General Hospital, National Defense Medical Center, Taipei 114, Taiwan; ^3^Division of Nephrology, Department of Internal Medicine, Yonghe Cardinal Tien Hospital, 80 Zhongxing Street, Yonghe District, New Taipei City 23445, Taiwan

## Abstract

At the early stage of chronic kidney disease (CKD), the systemic mineral metabolism and bone composition start to change. This alteration is known as chronic kidney disease-mineral bone disorder (CKD-MBD). It is well known that the bone turnover disorder is the most common complication of CKD-MBD. Besides, CKD patients usually suffer from vascular calcification (VC), which is highly associated with mortality. Many factors regulate the VC mechanism, which include imbalances in serum calcium and phosphate, systemic inflammation, RANK/RANKL/OPG triad, aldosterone, microRNAs, osteogenic transdifferentiation, and effects of vitamins. These factors have roles in both promoting and inhibiting VC. Patients with CKD usually have bone turnover problems. Patients with high bone turnover have increase of calcium and phosphate release from the bone. By contrast, when bone turnover is low, serum calcium and phosphate levels are frequently maintained at high levels because the reservoir functions of bone decrease. Both of these conditions will increase the possibility of VC. In addition, the calcified vessel may secrete FGF23 and Wnt inhibitors such as sclerostin, DKK-1, and secreted frizzled-related protein to prevent further VC. However, all of them may fight back the inhibition of bone formation resulting in fragile bone. There are several ways to treat VC depending on the bone turnover status of the individual. The main goals of therapy are to maintain normal bone turnover and protect against VC.

## 1. Introduction

CKD is a complex disease which impacts millions of people. Progression of CKD is associated with a number of serious complications, including hypertension, hyperlipidemia, anemia, hyperkalemia, mineral bone disorder, and cardiovascular disease. CKD patients always experience both renal bone disease and VC [1–5] and especially experience the more severe complications of these two conditions while on hemodialysis [6]. Compared with the non-CKD population, the cardiovascular death rate is at least 10 times higher and in young subjects this risk is more than 100-fold [7]. When the estimated glomerular filtration rate (eGFR) is less than 60 mL/min/1.73 m^2^, the cardiovascular risk is increased [8, 9]. A 30% decrease in eGFR is associated with a 20–30% increased risk of major cardiovascular events and all-cause mortality in patients with CKD [10].

Examination of CKD patients who have VC reveals two different but overlapping arterial pathologies: atherosclerosis and arteriosclerosis [11]. Atherosclerosis is primarily an intimal disease, with patchy plaques that spread and occur preferentially in medium-sized arteries. By contrast, arteriosclerosis is calcification of the media layer, which usually occurs along the elastic lamina which may lead to increased arterial stiffness [7].

In CKD patients, dysregulation of calcium and phosphate metabolism is the main contribution to VC. Elevated calcium and phosphate have direct effects on vascular smooth muscle cells (VSMCs). In turn, the VSMCs stimulate osteogenic/chondrogenic differentiation, vesicle release, apoptosis, loss of inhibitors, and extracellular matrix degradation to drive VC [12].

**Figure 1:**
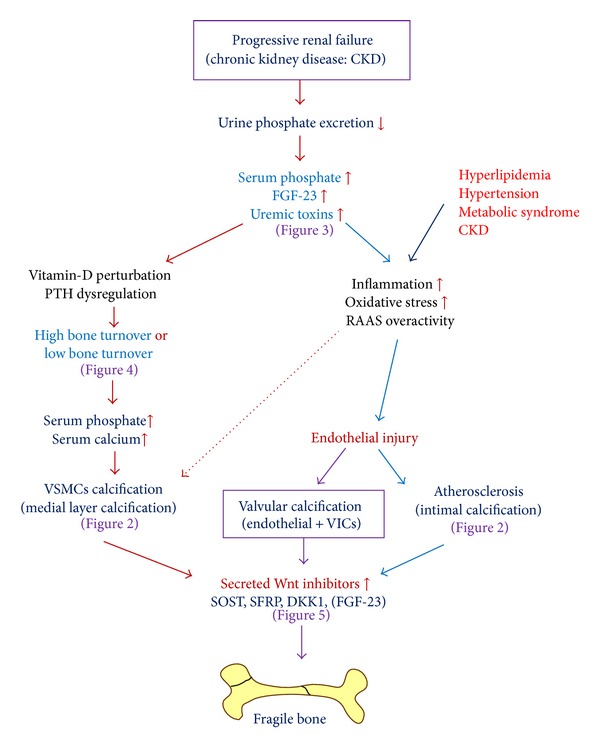
Scheme for possible mechanisms of vascular calcification in CKD. Vascular calcification is a prominent feature of arterial disease in CKD and may have an impact on cardiovascular mortality through modulating both arteriosclerosis (arterial stiffening) and atherosclerosis. In CKD, abnormal mineral metabolism, predominantly hyperphosphatemia and hypercalcemia, facilitates the progression of the active process of osteogenesis in vascular smooth muscle cells (VSMCs) resulting in arteriosclerosis calcification. However, the disruption of endothelial-derived relaxing factors may signal an early stage in atherosclerosis. Hyperlipidemia, hypertension, metabolic syndrome, and CKD are the major causes of endothelial injury, partly through increase of inflammation or oxidative stress. Major cell players are endothelial cells (or valve interstitial cells; VICs), leukocytes, and intimal smooth muscle cells (SMC). Focal calcification within atherosclerotic plaques is due to both active (osteogenic) and passive (cellular necrosis) processes. The phenotypic osteocyte in calcified vessels/valves may secrete Wnt inhibitors, which may fight back inhibition of bone formation.

Twenty years ago, a meaningful inverse association between bone mineral density and aortic calcification was suggested [13]. Some reports have pointed to a perplexing connection between VC and impaired bone metabolism and increased mortality [13–17]. Moreover, severe VCs are likely to be related to an increased frequency of nontraumatic fractures in both the general population and dialysis patients [17]. Usually, osteoporosis and VC are considered to be disorders of aging. However, a new study suggests that besides aging, there are other biological factors influencing the connection between VC and impaired bone metabolism, which contribute to arteriosclerosis and osteoporosis [18]. This review discusses both the pathophysiology of VC and its relationship to impaired bone metabolism in CKD patients (Figure 1).

## 2. Histoanatomic Classification of Cardiovascular Calcification in CKD

**Figure 2:**
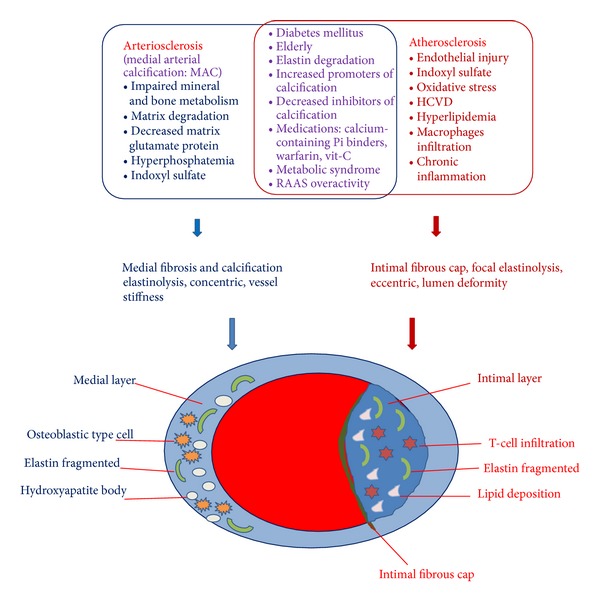
Risk factors associated with medial arterial calcification (MAC) versus atherosclerosis in CKD (above). Simplified histopathology pictures of the MAC and atherosclerotic calcification (below). Disordered mineral metabolism in CKD with its associated characteristics of hyperphosphatemia, hypercalcemia, and renal osteodystrophy, as well as vitamin D perturbation and klotho deficiency, increases the risk for MAC. Oxidative stress and chronic inflammation in uremia also accelerate atherosclerosis. A number of risk factors can drive both pathologies. Both atherosclerotic calcification and medial calcification stiffen arterial conduit vessels, impairing heart function. The eccentric remodeling of atherosclerotic calcification also reduces lumen diameter and predisposes to acute thrombosis.

Depending on the site, there are two main kinds of calcification: vascular wall calcification and cardiac valve calcification. Furthermore, VC can be divided into atherosclerosis and arteriosclerosis. This means it could be only one disease or two distinct ones existing at the same time. In CKD patients, most patients have two kinds of calcification simultaneously and overlapping pathological processes [7] (Figure 2).

### 2.1. Atherosclerosis

The intimal layer consists mainly of endothelial cells with some subendothelial connective tissues. Inflammation, thickening, and calcification of the intimal layer are called atherosclerosis [6]. The characteristics of atherosclerosis are lipid-laden plaques, primarily limited to the tunica intima of the arterial wall, and microinflammation of the atherosclerotic plaque [19]. Atherosclerotic burden starts at a young age and continually progresses, occurring mostly in medium-sized arteries and near arterial branch points. Atherosclerosis is patchy and focal in its distribution and predominantly affects medium-sized conduit arteries such as the epicardial coronary, carotid, iliac, and femoral arteries [20].

### 2.2. Arteriosclerosis or Medial Arterial Calcification (MAC)

The medial layer contains VSMCs and an elastin-rich extracellular matrix. The deeper layer of calcification in the media elastic matrix of the arterial wall is termed arteriosclerosis [16]. Arteriosclerosis is the fibrosis, thickening, stiffening, and calcification of the medial arterial layer in the large- and medium-sized arteries and may lead to left ventricular hypertrophy [16]. In contrast with the focal and patchy distribution of atherosclerosis, arteriosclerosis effects the tunica media in a diffuse contiguous style [19]. Several studies have demonstrated that medial artery calcification (MAC) is associated with major changes in the microstructure of the arterial wall, including increased extracellular matrix deposition, elastin degradation, and apoptotic bodies. Unlike atherosclerosis, lipid-laden plaques are not a specific feature of MACs [19].

Recent studies suggest that altered mineral metabolism, such as high or low bone turnover disorders in CKD, may promote arteriosclerosis, and inflammation is not characteristic of MAC lesions [21], whereas increased inflammation and oxidative stress, such as hyperlipidemia, hypertension, metabolic syndrome, and CKD, may contribute to atherosclerosis as well. Atherosclerosis and MAC can usually be easily distinguished as spotty calcifications versus linear tram-track calcifications on plain radiographs [22].

### 2.3. Cardiac Valve Calcification

Calcification of the cardiac valve leaflets can change the mechanical properties of the tissue and cause stenosis [6]. Calcification of the valves is commonly associated with hyperlipidemia and aging and is the most common pathology seen in excised native valves [23]. Because of the long-standing action of mechanical stress and proinflammatory factors, the aortic valve is usually affected by dystrophic calcification [24, 25]. In the early stages of valve calcification, the lesion includes abundant subendothelial lipids and extracellular matrix, with displacement of the elastic lamina. As the disease worsens, lipids, extracellular matrix, osteoblastogenesis, and the presence of calcifying vascular cells (CVCs) [26–28] are increased, with evidence of breakdown and disarrangement of the elastic lamina [29]. All of these chronic inflammatory factors will cause circulating osteoprogenitors, endothelial-mesenchymal transition, and annular chondrocytes and result in cardiac valve calcification. On the other way, the chronic inflammation will promote bone turnover disorders to stimulate vascular wall calcification, but not cardiac valve calcification [30].

Potential roles for calcific aortic valve disease in the pathogenesis are lipoprotein retention and signaling, oxidative stress, and renin-angiotensin system activation [31]. The calcification of the aortic valve stroma does not exhibit similar evident features of atherosclerotic changes [25]; however, it may be proof that the early stages of calcification of the valve cusps proceed by different mechanisms and may predict atherosclerosis progression in coronary arteries [32]. Similarly, the mitral valve can be found to have calcium deposits and the annulus of the valve to contain cartilaginous metaplasia [33].

## 3. Pathophysiology of VC

There are many factors impacting VC, which is a precise complex process. In CKD patients, these factors often include abnormal activities and serum levels, such as hyperphosphatemia, which is a major important contributor to VC [34]. Calcification starts after release of vesicular structures from VSMCs, which contain hydroxyapatite [35]. The calcification process includes active and passive processes. The active process is the transformation of VSMCs to an osteogenic/chondrogenic phenotype that promotes the release of the vesicular structures. Mineralization in these structures is stimulated by osteoblastic proteins. The key transcription factors involved in osteoblast differentiation and translation of bone proteins, such as alkaline phosphatase and bone morphogenetic protein 2 (BMP2), are all components of the osteoblastic transformation of VSMCs [36]. On the other hand, the passive process involves mineral precipitation from the extracellular fluid surrounding the VSMCs in the vascular walls [18]. It has been recognized recently that there are many promoters as well as inhibitors of calcification that may have general or local effects on VC.

### 3.1. Calcification Promoting Factors

The relationship of vascular calcification between promoters and inhibitors is really unclear, but they could counterbalance each other in the general population. However, in CKD patients, inhibitory systems, such as matrix glutamate protein (MGP), pyrophosphate, and fetuin-A [19], are overwhelmed by a multitude of promoters agents that induce VSMC damage and cell death resulting in vascular calcification [37].

#### 3.1.1. Calcium/Phosphate Homeostasis

*Normal Phosphorus Metabolism*. Fibroblast growth factor 23 (FGF23), klotho, parathyroid hormone (PTH), and 1,25-dihydroxyvitamin D are basic regulators of phosphorus metabolism. About 50–80% of dietary phosphate is absorbed by the gastrointestinal tract via the sodium-phosphate cotransporters, NaPi-2b (SLC34A2) and PiT-1 (SLC20A1), or by passive paracellular pathways [38, 39]. FGF23 and PTH increase kidney phosphate excretion by downregulating the NaPi-2a (SLC34A1) and NaPi-2c (SLC34A3) channels in the proximal tubules [40–42]. Klotho is a transmembrane protein that acts as an essential cofactor for FGF23 at its receptor but can also promote phosphaturia independently of FGF23 by inactivating NaPi-2a [43]. The 1,25-dihydroxyvitamin D enhances intestinal phosphate absorption by increasing the expression of NaPi-2b and also regulates PTH and FGF23 [44].

*Dysregulation of Phosphate and Calcium Homeostasis in CKD*. In the past decade, hyperphosphatemia has been proved as an independent factor for cardiovascular events and decreasing bone mineral density [45, 46]. Abnormal phosphate metabolism occurs early in patients with an eGFR less than 60 mL/min, as evidenced by increased serum FGF23 levels, which occur before PTH increases [47, 48]. FGF23 also directly contributes to active vitamin D deficiency by inhibiting 1*α*-hydroxylase in the kidney [49]. In addition, increased FGF23 influences other systems as well by promoting left ventricular hypertrophy and deteriorating renal function, thereby increasing mortality [50].

Calcium regulation also relies on the VSMC phenotypic circumstance [51, 52]. Differentiated contractile VSMCs can display high levels of the voltage-activated L-type calcium channels, which take up extracellular calcium, and the sarcoplasmic reticulum intracellular calcium release channel, the ryanodine receptor. However, VSMCs endure phenotypic modulation, which coincides with the beginning of osteogenic/chondrogenic differentiation. Increased expression of the low-voltage-activated T-type channels and downregulation of L-type channel expression occur in VSMCs. The ryanodine receptor is also downregulated, which is compensated for by increased expression of other sarcoplasmic reticulum ion pumps [51]. Thus, changes in both intracellular and extracellular calcium pools are likely to dramatically influence VSMC function [12].

Phosphate can lead to changes in the vessel wall by promoting calcification, and this calcification is not simply the result of calcium and phosphorus precipitation from the circulation [53]. Hyperphosphatemia may stimulate endothelial cells (ECs) to form microparticles. Then, the microparticles thrown off by the ECs will decrease the secretion of annexin II, reduce angiogenesis, increase the production of reactive oxygen species (ROS), enhance inflammation, and result in apoptosis of the ECs [54]. VSMCs cultured in phosphorus concentrations ranging from 1.6 to 3 mmol/L (5 to 9.3 mg/dL) fail to express smooth muscle proteins and begin expressing genes recognized as markers of osteoblasts such as Runx2/Cbf*α*1, osteopontin, and alkaline phosphatase [55, 56]. Resembling normal bone formation, these transdifferentiated VSMCs deposit minerals in the extracellular matrix via matrix vesicles [36, 57] in a tightly regulated sequence, and the degree of calcification corresponds to the phosphate dose [55]. Additionally, a study showed that Stanniocalcin 2 can inhibit phosphate-induced ectopic calcification in VSMCs and may play a role in the treatment of VC [58].

#### 3.1.2. Osteochondrogenesis

Hyperphosphatemia induces calcification by upregulating mRNA expression for osteogenic factors including BMP 2, Runx2/Cbf*α*1, Msx2, and osteocalcin [59]. Runx2 is an important transcription factor in osteoblastic and chondrocytic differentiation, causing the expression of major bone matrix components such as osteocalcin, type I collagen, and osteopontin (OPN). Hyperphosphatemia also leads the activation of the Wnt/*β*-catenin signaling pathway by the translocation of *β*-catenin into the smooth muscle cell nucleus, increasing the expression of direct target genes such as cyclin D1, axin 2, and VCAN/versican [59].

In fact, the role of BMP and Wnt signaling on VC is still unclear [60]. Both proteins regulate bone mass by promoting osteogenesis by stimulating Runx2 gene expression [59], but the molecular interactions between these pathways in osteogenesis and bone formation are not completely defined [61]. Therefore, hyperphosphatemia can induce osteogenic/chondrogenic phenotype changes, the ultrastructural characteristics of which are the formation of bone matrix vesicles containing apatite and calcifying collagen fibrils on the surface of VSMCs [57]. Furthermore, these vesicles almost certainly act as early nucleation sites for calcification in the vascular wall [59].

#### 3.1.3. Apoptosis

Hyperphosphatemia has been shown to induce VSMC apoptosis [36, 62]. Some studies report that VSMCs are unable to adapt to the high-phosphate environment then become bone-like cells and follow the cell-death route [62]. As VSMCs undergo apoptosis, they excrete large numbers of apoptotic bodies from their cell surface. Both matrix vesicles and apoptotic bodies promote extracellular calcification by acting as nucleation sites for mineral deposition in the extracellular matrix. As a result, calcium and phosphorus deposition occurs [57, 63].

#### 3.1.4. Circulating Calciprotein Particles

Calciprotein particles consisted of fetuin-A, albumin, and other acidic proteins as well as calcium and phosphate. In Heiss et al., fetuin-A was important for the formation and stabilization of the calciprotein particle bodies [64]. Serum levels of these particles seem to rise as renal function deteriorates, with the highest level of particles detected in dialysis patients. Previous studies showed that the concentration of calciprotein particles may be a more sensitive measure of extraosseous calcification than total fetuin-A serum concentrations in dialysis patients [65].

#### 3.1.5. Matrix Degradation/Modification

The collagens, elastins, fibronectins, heparan sulfates, proteoglycans, and chondroitin sulfate proteoglycans frame the complex, highly structured extracellular matrix to encompass VSMCs [66–68]. Elastin is secreted from VSMCs as the soluble monomer, tropoelastin [69]. In people with CKD and hyperphosphatemia and in animal models, the accumulation of linear mineral deposits along the arterial elastic lamina is a key feature of the predominant type of arterial medial calcification [66–68].

Recent studies suggest that elastin fragmentation may enhance arterial calcification in end-stage renal disease (ESRD). Elastin degradation causes the extracellular matrix to have a higher affinity for calcium and facilitates epitactic growth of hydroxyapatite along the elastic fibers [44]. Elastin fragments bind not only to elastin laminin receptors located on the surface of VSMCs, but also through transforming growth factor-*β* signaling [69], which can promote proliferation and upregulate Runx2, leading to osteogenic differentiation [70, 71].

### 3.2. A Decrease in Factors That Inhibit VC

There are many endogenous factors that inhibit calcification of the arterial wall under healthy conditions, such as matrix glutamate protein (MGP), fetuin-A, and pyrophosphate.

#### 3.2.1. Decreased Matrix Glutamate Protein

MGP synthesized by VSMCs [72] is found at the interface between normal tissue and the mineralized lesions of calcified arteries in patients with CKD or diabetes [73]. The precise mechanism of MGP is unclear; it may inhibit BMP2 and BMP4 to block the induction of the VSMC osteoblastic phenotype, or it may directly bind to hydroxyapatite [74]. Previous studies have shown that vitamin K-dependent *γ*-carboxylation of glutamate residues is mandatory for MGP's ability to chelate minerals and inhibit calcification [75, 76]. In addition, MGP deficiency may cause severe arterial calcifications [75, 77].

#### 3.2.2. Decrease in Fetuin-A

Fetuin-A, a calcium-binding glycoprotein present at high concentrations in human blood, is secreted by hepatocytes and safely cleared by the liver. Fetuin-A is principally responsible for inhibiting spontaneous mineral precipitation from serum [78]. It inhibits de novo calcium phosphate precipitation [79] and also inhibits calcification within VSMCs, preventing vesicular-mediated precipitation of calcium phosphate [80]. Many studies show that low serum fetuin-A levels are linked to the progression of atherosclerosis, aortic calcification, and increased cardiovascular disease mortality in ESRD patients [81–84].

#### 3.2.3. Decreased Pyrophosphate (PPi)

Pyrophosphate concentrations are lower in dialysis patients compared to healthy controls and are one of the reasons why dialysis patients are more susceptible to VC [85]. Pyrophosphate can directly reduce hydroxyapatite formation within VSMCs as well as decrease nanocrystal formation [63]. The tissue nonspecific alkaline phosphatase hydrolyzes pyrophosphate to produce inorganic phosphorus, which will enhance calcifying VSMCs to undergo osteogenic differentiation and worsen VC [19, 86].

### 3.3. Inflammation and Reactive Oxygen Species

Inflammation and reactive oxygen species are two additional factors associated with VC. Inflammation may promote VC by releasing “tumor necrosis factor *α*,” which triggers the Wnt signaling pathway, resulting in osteogenic differentiation of VSMCs [18, 87–90]. In addition, several other factors related to oxidative stress may be involved. Among them, hydrogen peroxide has been reported to stimulate Cbf*α*-1 directly [91] as well as BMP2, which increases osteoblastic differentiation of calcifying cells and may also reduce the expression of MGP [92].

### 3.4. Downregulation of the PTH Receptor

PTH and PTH-related peptide (PTHrP) may act as mediators of VC. Both PTH and PTHrP avoid VSMC calcification in a dose-dependent manner by inhibiting alkaline phosphate activity [93]. While PTH fragments 1–34 were shown to inhibit VC in an animal study [94], PTH 7–84 might increase the risk of VC [95]. In CKD patients, downregulation of the PTH receptor in VSMCs attenuates the protective effects of PTH on VC [96]. However, no data as yet link PTH to Cbf*α*-1 expression in VSMCs [97]. But, other studies showed that PTH levels are proportional to calcification scores [98]. In contrast, other researches demonstrated that PTH cannot induce VC but have a synergistic effect with phosphate, probably because of an indirect association with bone remodeling and osteoclastic activity with a damaging result [99].

### 3.5. Magnesium

Magnesium can replace Ca in carbonated hydroxyapatite and destabilize the crystal structure of hydroxyapatite; thus, it may contribute to its potential solubility. A study demonstrated that magnesium inhibited the transformation of VSMCs into osteoblast-like cells as well as hydroxyapatite crystal formation [100]. Several clinical studies suggested inverse relationships between serum magnesium levels and VC [101].

### 3.6. Role of Vitamins in Calcification

#### 3.6.1. Vitamin K

There are two forms of vitamin K: vitamin K_1_ (phylloquinone, mainly derived from vegetables) and vitamin K_2_ (menaquinone, mainly derived from fermented food such as cheese). Vitamin K_2_ has a specific role in osteoblast, chondrocyte, and VSMC function [101]. In general, vitamin K_2_ carboxylates extrahepatic Gla proteins such as MGP and osteocalcin [102]. MGP, a vitamin K-dependent protein, could be activated by *γ*-carboxylation, whereas undercarboxylated proteins are inactive. In a human study, vitamin K_1_ could be converted into vitamin K_2_ by the intrinsic enzyme, UbiA prenyltransferase domain-containing 1 [103]. In another pilot study in dialysis patients, VC was inversely associated with plasma concentrations of desphospho-carboxylated MGP, and the plasma levels of desphospho-undercarboxylated MGP could be significantly reduced (−27%) by daily supplementation with vitamin K_2_, suggesting a shift to the active, carboxylated MGP isoform within the vessel wall [104].

Warfarin is commonly prescribed to patients with CKD because of their higher incidence of atrial fibrillation [105]. Use of warfarin results in a phenotype similar to that seen in MGP-deficient mice [106]. This condition may disrupt vitamin K-dependent gamma-carboxylation (activation) of MGP. Therefore, warfarin is a strong risk factor for VC, not only in dialysis patients [107, 108], but also in the general population [97]. Additionally, unfractionated heparin will increase osteoclast activity and promote VC [109].

#### 3.6.2. Vitamin C (Ascorbic Acid: AA)

The biochemical role of AA has been described for several decades [110]. From the molecular mechanisms of VC, AA promotes the pathogenesis of this process. AA plays an essential role in different biological functions, such as mesenchymal [111] and chondrocyte differentiation [112].

AA exposure will increase the phenotype of important genes implicated in bone mineralization as well as VC, such as collagen II, Cbf*α*1/RUNX2, Sox 9, and collagen X. Moreover, AA heightened Erk activation, whereas Erk inhibition weakened AA-induced differentiation [112]. Furthermore, AA also enhances calcitriol synthesis through upregulation of the vitamin D receptor. Therefore, AA may increase vitamin D receptor-dependent genomic responses to calcitriol, leading to promotion of terminal differentiation [113]. Ciceri et al. found synergistic effects of AA and phosphate in promoting VC. This role of AA may be to act as a cofactor in intracellular collagen biosynthesis, bone mineralization, expression of key genes (Cbf*α*/RUNX2), and extracellular calcium deposition in osteoblast-like cells [114].

#### 3.6.3. Vitamin E

How vitamin E induces VC is unclear, but in bone remodeling vitamin E enhances the expression of dendritic cell-specific transmembrane protein (DC-STAMP) which increases the size of osteoclasts, their number of nuclei, and bone-resorbing ability [115]. Vitamin E also stimulates RANKL secretion and mergers of osteoclast cells. One study showed that the vitamin E isoform enhances protein kinase signaling, leading to phosphorylation and stimulating the transcriptional regulator, microphthalmia-associated transcription factor to bind to the promoter of the gene encoding DC-STAMP (Tm7sf4), thereby promoting VC [116].

#### 3.6.4. Role of Vitamin D

Treatment with high doses of calcitriol or toxic doses of vitamin D_3_ causes vascular and soft-tissue calcifications [117–120]. Mizobuchi et al. demonstrated that calcitriol and doxercalciferol, but not paricalcitol, induced severe medial calcification in 5/6 nephrectomized rats, despite using doses that efficiently lowered elevated levels of PTH [121]. Calcitriol induces RANKL expression on osteoblastic cells. RANKL enhances a signal for osteoclast differentiation and activation through its receptor RANK present on osteoclast progenitors and promoted osteoclast function [122]. By comparing vitamin D receptor-null (VDR−/−) and VDR+/+ mice, high-dose calcitriol was found to induce VC regardless of vitamin D receptors in the aortas. Therefore, vitamin D promotes VC due to its systemic effect [123]. VSMCs manifest vitamin D receptors [124] and high calcitriol doses induce matrix mineralization of VSMCs in vitro [93]. It is most likely that calcitriol may improve bone health but deteriorate vascular calcification through increase mineral burden in CKD. However, the physiological function of vitamin D receptor activation in VSMCs is inhibitory to matrix mineralization through its stimulation of smooth muscle differentiation and repression of osteoblastic transition [125].

The effects of vitamin D on calcification of VSMCs can proceed in two opposite (contradictory) directions. The first is the procalcific effects, which include direct influences on VSMCs, promoting VC by raising phosphate and calcium levels, and oversuppression of PTH leading to adynamic bone disease and low bone turnover. In contrast, the other has protective effects, which are pleiotropic anti-inflammatory and immunomodulatory effects on the cardiovascular system [126], inhibiting the production of renin and myocyte proliferation [127] and preventing hyperparathyroidism. Generally speaking, in CKD patients with low levels of nutritional vitamin D, supplementation with low-dose vitamin D has been proved to reduce mortality [128].

### 3.7. Role of Aldosterone

**Figure 3:**
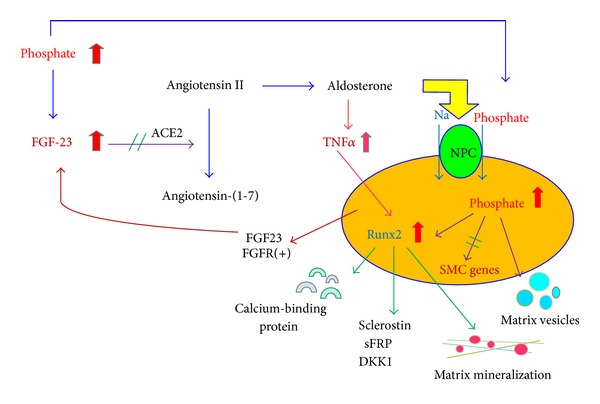
Factors related to the osteogenic transdifferentiation of vascular smooth muscle cells in chronic kidney disease (CKD). Hyperphosphatemia stimulates the secretion of FGF-23 from osteocytes in the bone, which inhibit the activity of angiotensin-converting enzyme 2 (ACE2). FGF23 blocks the conversion of angiotensin II into angiotensin (1-7). Therefore, angiotensin II will enhance the production of aldosterone. Phosphate and calcium stimulate the Na-Pi cotransporter, and aldosterone also contributes to activate the Na-Pi cotransporter, resulting in increased phosphate entrance into VSMCs. In addition, aldosterone accentuates the inflammatory status in part by TNF*α*. Both oxidative/inflammatory status and increased intracellular phosphate levels promote VSMCs to transdifferentiate into phenotypic osteoblast cells, which causes the ossification of the vascular wall to progress. As a whole, the calcified vessels have more prominent bone formation characteristic than bone resorption ones. In addition, osteogenic cells may secrete sclerostin (SOST) and FGF23. The secreted FGF23 from the calcified vessel may contribute further increased FGF23 serum levels.

Elevated serum phosphate may stimulate FGF23 production, which in turn may inhibit the angiotensin converting enzyme (ACE) 2, resulting in increased angiotensin II (Figure 3) and aldosterone levels in CKD [129]. VSMCs express mineralocorticoid receptors and their activation by mineralocorticoids promotes VC [130, 131]. High-plasma aldosterone concentrations are related to vascular stiffening, vascular damage, and atherosclerosis [132–134]. Aldosterone could directly regulate the type III sodium-dependent phosphate transporter-phosphate transporter 1 (Pit-1) in vitro. Pit-1 is required for the subsequent TNF*α*/Msx2 cascade, causing chondroosteogenic transformation as well [135]. In addition, aldosterone also upregulates vascular TNF*α* [136]. TNF*α* promotes the differentiation of VSMCs into cells that are characteristic of chondroblastic/osteoblastic cells [137]. TNF*α* stimulates the expression of the chondroosteogenic transcription factors osterix and Cbf*α*1/Runx2. This step includes the transcription factors Nf-*κ*B and Msx2 [138]. Cbf*α*1/Runx2 is another key factor in triggering VC [139].

### 3.8. MicroRNAs: New Players in VC

MicroRNAs are small, noncoding, single-stranded RNAs that function in transcriptional and posttranscriptional regulation of gene expression [140]. MicroRNAs bind to complementary sequences on target mRNAs, usually leading to gene silencing through translational repression or target degradation [141]. The role of microRNAs in cardiovascular biology is currently under intense investigation, and specific microRNAs have been associated with various cardiovascular disorders, including vascular remodeling, cardiac hypertrophy, heart failure, and postmyocardial infarction remodeling [142]. One study stated that specific miR-143 and miR-145 in VSMCs were reduced in patients with CKD and/or atherosclerosis. By contrast, inflammatory miR-223 and miR-126 were elevated in patients with more advanced CKD [143]. However, increasing evidence shows that the reduction of microRNAs may play a pivotal role in increasing Runx2 and decreasing myocardin, contributing to VC [144].

### 3.9. Indoxyl Sulfate

CKD patients accumulate uremic substances in their body because of impaired kidney function. These uremic substances, called uremic toxins (UTx), have been reported to injure various organs. Indoxyl sulfate (IS) is one of the uremic toxins and is derived from tryptophan. It is excreted mainly from the proximal renal tubules into urine. Decrease renal clearance as occurs in chronic renal failure will increase IS levels in the blood, so levels are approximately 30 times higher in patients with CKD stages 4-5 (not yet on dialysis) patients and 80 times higher in patients before initiation of dialysis than in healthy persons [145]. A study of Dahl salt-sensitive hypertensive rats proved that IS significantly increased aortic calcifications [6], but IS concentration is also positively related to aortic calcification in patients with CKD [146].

In addition, Mozar et al. confirmed that IS directly inhibits osteoclast differentiation and activity [147]. Furthermore, Kim et al. illustrated that IS inhibits osteoblast differentiation and induces apoptosis via the caspase (cysteine aspartate protease) pathway [148, 149]. Therefore, as a bone toxin, IS may deteriorate the outcomes of low bone turnover diseases and attenuate the chemical composition of bone in patients with CKD. This mechanism may explain the increased incidence of hip fractures in CKD patients [150]. Oral administration of the indole-absorbing agent, AST-120, prevents the progression of VC [151] and improves the low bone turnover status in patients with CKD [145].

## 4. Bone-Vascular Axis and VC

### 4.1. RANK/RANKL/OPG and VC

It is obvious that impaired bone metabolism has an important role in the development of VC [18]. More and more researchers now advise that VC, like bone remodeling, is an actively regulated process, including both stimulatory and inhibitory processes [152]. There are three key elements that influence the bone formation process: receptor activator of NF-*κ*B (RANK), receptor activator of NF-*κ*B ligand (RANKL), and osteoprotegerin (OPG). RANK, a type I membrane protein on the surface of osteoclast cells, is involved in osteoclast cell stimulation when bound with receptor activator of NF-*κ*B ligand (RANKL) produced by osteoblasts [153].

In addition, mounting evidence suggests that the RANK/RANKL/OPG triad is involved in bone metabolism and may be important in VC. OPG, RANKL, and RANK exist in extraosseous calcifications such as atherosclerotic calcifications and cardiac valve calcifications. Also, their relative expression levels are different depending on the stage of the disease [154, 155]. RANKL/RANK signaling not only regulates the formation of multinucleated osteoclast cells from their precursors, but also influences their activation and survival during normal bone remodeling and in a variety of pathologic conditions [156]. OPG, the other protein secreted from osteoblast cells, is a potent inhibitor of osteoclast differentiation and protects the skeleton from excessive bone resorption by acting as a decoy receptor for RANKL and preventing RANKL from binding to its receptor, RANK [153]. Thus, the RANKL/OPG ratio is a significant determinant of bone formation.

Panizo et al. stated that RANKL directly increased VSMC calcification by binding to RANK and stimulating BMP4 secretion by the alternative NF-*κ*B pathway [157]. Thus, RANK/RANKL may be crucial in stimulating VC, whereas OPG inhibits VC. OPG appears to be the molecular link between bone resorption and VC, which may help recognize the close relationship between atherosclerosis and osteoporosis in postmenopausal women [32]. Shargorodsky et al. stated that serum OPG level is an independent predictor of early cardiovascular events in osteoporotic postmenopausal women [158].

### 4.2. Altered Bone Turnover and VC in Patients with CKD

The bone has a complex connection with the vascular system. Both have similar and mutual changes in mineralization, a situation called the bone-vascular axis [159]. In the past 20 years, it was repeatedly reported that the association between bone fragility and VC was because of a significant inverse correlation between bone mineral density and aortic calcification [13]. But, this correlation is poorly understood and underlying relationships have not yet been well-characterized. Besides, CKD is usually thought to represent a state of low bone turnover, which reduces the bone's ability to buffer mineral metabolites such as calcium and phosphate, which promotes VC [160]. Therefore, low bone turnover is associated with coronary artery calcium (CAC) score progression in hemodialysis patients [161].

Some initial studies also revealed that low bone turnover or mineralized bone volume is inversely related to the degree of coronary artery calcification and vascular stiffness [162]. Rodríguez-García et al. analyzed hemodialysis patients and found that calcification in the large and medium arteries is associated with a higher possibility of vertebral fractures [4]. This study showed that both VC and vertebral fractures were associated with increased mortality among research samples [4].

**Figure 4:**
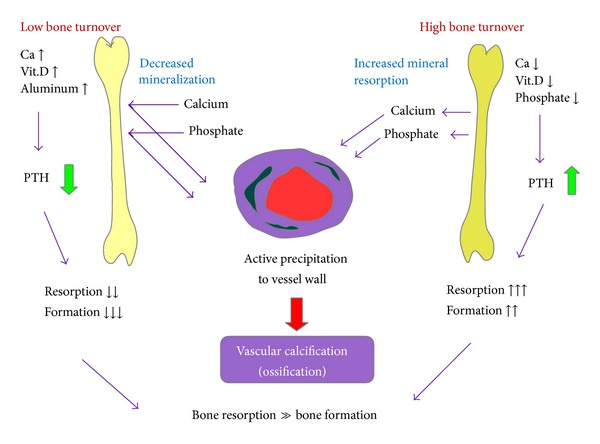
Bone turnover and vascular ossification in chronic kidney disease (CKD). Basically, bone cells have less vitality in patients with CKD than in normal persons. Thus, low bone turnover is part of the innate character of CKD. High PTH serum levels will overcome the indolent bone cells and lead to high turnover bone disease with the characteristics of relatively higher bone resorption than bone resorption. The high turnover status in SHPT can induce increased bone demineralization, which will increase calcium and inorganic phosphate release from bone into circulation. In contrast, overtreatment of CKD patients with Ca-salts, VDRA, or aluminum may cause them to develop low turnover bone disorders and low serum PTH levels. In patients with low bone turnover status, the decreased bone mineralization makes it difficult for calcium and inorganic phosphate to enter into bone, resulting in increased serum calcium and inorganic phosphate. Both high and low bone turnover disorders are characterized by a relatively higher degree of bone resorption than bone formation, which may contribute to the elevated serum calcium and inorganic phosphate levels, and aggravate vascular calcification/ossification.

However, the relationship between low bone turnover and VC remains unclear [98]. Recent publications examining low or even high bone turnover discovered that VC is not influenced by bone turnover itself but is related to situations where bone resorption is greater than bone formation (Figure 4). These researchers found that VC can occur at any level of bone turnover [98]. As previously stated, serum phosphate may be one of the connections between bone turnover and VC. When bone turnover is low, as with adynamic bone, the amount of interchangeable calcium and phosphate is decreased, leading to higher concentrations associated with intake. Furthermore, bone resorption is more prominent than bone formation, interfering with the buffering function of the skeleton for extra phosphate. By contrast, when high bone turnover is present as in secondary hyperparathyroidism, a lot of phosphate is released from bone and, again, the reservoir function of the skeleton is destroyed [18]. Therefore, correcting the balance in bone, either high or low, will protect against the progression of VC [161].

### 4.3. Calcified Vessel Impaired Bone Metabolism

**Figure 5:**
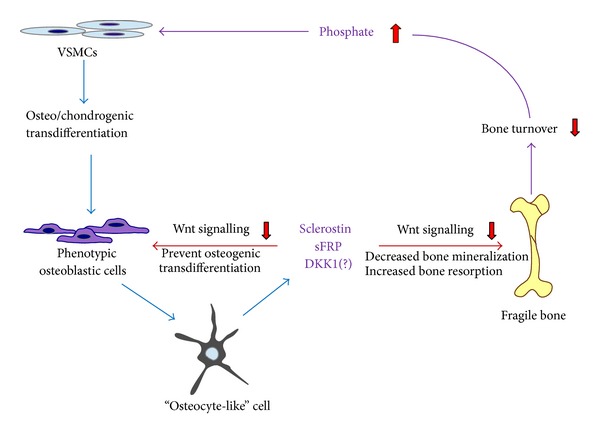
Calcified vessels on bone turnover in chronic kidney disease (CKD). The phenotypic osteoblast/osteocyte in calcified vessels may secrete sclerostin (SOST), secreted frizzled-related protein (sFRP), and Dickkopf-related protein 1 (DKK1), which prevent further calcification of the affected vessel. The secreted SOST and sFRP will process autocrine or paracrine effects to inhibit Wnt signaling effects on osteogenic transdifferentiation of VSMCs, which will prevent further calcification of the vessel wall. As the SOST and sFRP secreted from calcified vessel are released into circulation, they may inhibit Wnt signaling in osteoblast in the bone. This inhibition of bone osteoblasts reduces bone accretion and turnover, resulting in a fragile bone, which may also contribute elevated serum inorganic phosphates. In addition, DKK1 enhances RANKL levels, and the increased RANKL : OPG ratio activates osteoclast activity, leading to the increase of bone resorption.

VC and impaired bone metabolism, the important causes of mortality and morbidity, are common in patients with CKD or osteoporosis, and in those who are aging [163]. The Wnt signaling pathway is a complicated network of several proteins that can regulate normal physiologic bone formation processes [164]. The consequence of Wnt signaling in bone is mediated by stimulation of stem cells and proliferation of preosteoblasts, induction of osteoblastogenesis, inhibition of osteoblast and osteocyte apoptosis, and attenuation of osteoclastogenesis [165, 166]. Thus, the physiological mechanisms of Wnt signaling lead to both formation and antiresorption benefits at the same time [167]. The effect of Wnt signaling depends on a transmembrane receptor complex composed of the frizzled receptor and the low-density lipoprotein receptor-related protein- (LRP-) 5 or LRP-6 coreceptors [165, 166]. Recent evidence supports the notion that there are inhibitors associated with the Wnt signaling pathway, such as sclerostin, secreted frizzled proteins 2 and 4, and Dickkopf-related protein-1 (DKK-1), that enhance osteoclast function and link VC and bone loss [168, 169] (Figure 5). Pinzone et al. stated that DKK-1 increases the osteolytic activity and decreases osteoblast differentiation [170].

Sclerostin, a glycoprotein inhibitor of osteoblastogenesis, is secreted by osteocytes and travels through osteocyte canaliculi to the bone surface where it binds to LRP-5 and LRP-6 coreceptors. Consequently, sclerostin prevents frizzled proteins from colonizing on bone and blocks Wnt signaling to reduce osteoblastogenesis and bone formation [164]. Then, sclerostin may have a negative feedback role in osteoblasts signaling at the onset of osteoid mineralization [171, 172]. Additionally, more recent evidence suggests that the Wnt signaling pathway not only plays an important role in bone metabolism, but also is involved in medial artery and aortic valve calcification [173–175]. Increased sclerostin expression has also been demonstrated in vascular and aortic valve calcification [175]. Because sclerostin secreted from either the osteoid or the calcified vessel may spread to the circulation, Drake et al. stated that bone marrow plasma and peripheral serum sclerostin levels were strongly correlated [176]. Thus, sclerostin may be a significant communicator between bone and vascular soft tissue calcification [46].

Some evidence suggests that ageing, diabetes, male gender, and low PTH levels are all associated with high circulating sclerostin levels [177–180]. Particularly, sclerostin levels also increase with the progression of CKD and correlate inversely with histological parameters of bone turnover, and osteoblast number and function in hemodialysis patients [172].

As described above, sclerostin spreads from the calcified vessel and may deteriorate bone structure and retard further mineralization [46, 175]. This may clarify why VC is negatively associated with bone density and positively related to fractures [4, 181]. To sum up, sclerostin may be a master mineralization regulator in VC [175] and impaired bone metabolism [182]. Therefore, sclerostin may play an important role in the bone-vascular axis [18].

## 5. Treatment of VC (Table 1)

### 5.1. Phosphate Binders

To improve clinical outcomes in CKD patients, one of the key therapeutic goals is to lower the phosphate load and maintain serum phosphorus levels within the normal range [183]. Another new strategic therapy in patients with CKD is to avoid high urinary phosphate excretion because high urine phosphate will induce kidney tubular injury and interstitial fibrosis. Thus, using phosphate binders in the early stages of CKD can not only lower urine phosphate, but also lower FGF23 in blood [184]. Several human trials in CKD support the notion that reducing serum phosphate levels can slow the mineralization of soft tissue [185, 186]. Treatments that reduce intestinal phosphate absorption include a low-phosphate diet and phosphate binders [183]. Phosphate binders are divided into two main types: calcium-containing binders and calcium-free binders. Currently, there are four types of non-calcium-based phosphate binders available: sevelamer, lanthanum carbonate, magnesium salts, and sucroferric oxyhydroxide (or PA21) [183, 187]. In addition, aluminum-containing agents have been commonly prescribed in the past but are not currently widely used because of their toxicity [183]. Although either calcium-containing binders or calcium-free binders have the same effects in decreasing serum phosphate levels, only the calcium-containing binder is believed to contribute to VC development and progression [186].

Sevelamer, the non-calcium-containing binder, is an ion-exchange resin with no concern for systemic accumulation and has pleiotropic effects, such as decreased LDL-cholesterol level, diminished inflammation, increased fetuin-A, and protection of cardiovascular function [188]. In contrast, lanthanum carbonate and magnesium salts are absorbed in the gut and excreted through bile for lanthanum and through the urine for magnesium [183].

Most patients on dialysis are currently taking many forms of phosphate binders. Clinical studies suggest that non-calcium-containing phosphate binders should be used in patients with a high risk for VC [185, 186]. In high turnover renal bone diseases, sevelamer, lanthanum, and PA21 can lower serum phosphate while avoiding hypercalcemia to decrease VC. Moreover, sevelamer and lanthanum can improve low bone turnover situations [187].

### 5.2. Vitamin D Receptor Agonists (Active Vitamin D and Nutritional Vitamin D)

Vitamin D is involved in regulating mineral metabolism; therefore, vitamin D deficiency is associated with early mortality in patients with CKD [6]. Vitamin D receptor agonists (VDRAs) are given to patients with CKD to correct the deterioration caused by hyperparathyroidism and vitamin D deficiency [6]. In hemodialysis patients, VDRA therapy can improve the survival rate, but its real mechanism is not fully understood [189].

From a study of animals with CKD, VDRA therapy significantly decreased aortic calcification because the benefits of VDRA therapy were associated with increasing levels of klotho and osteopontin [190]. Recent studies revealed that nutritional vitamin D supplementation can have long-term beneficial effects on cell proliferation and cardiovascular and immune disorders as well as on survival rates [191, 192]. Even in the presence of low bone turnover, nutritional vitamin D and low-dose active vitamin D could activate osteoblasts because 1*α*-hydroxylase is present in all three major bone cell types, including osteoblasts, osteoclasts, and osteocytes [193–195]. However, repletion of active rather than nutritional vitamin D will result in elevated 1,25(OH)_2_D levels, which will inhibit hepatic 25-hydroxylation, resulting in nutritional vitamin D deficiency [196, 197]. In addition, Mizobuchi et al. have stated that calcitriol and doxercalciferol induce severe medial calcification in rats with CKD, in spite of supplement doses that decreased the elevated levels of parathyroid hormone [121]. Therefore, a bimodal effect of vitamin D analogues can be postulated with regard to regulation of calcification [101].

### 5.3. Vitamin K

Vitamin K, a group of cofactor vitamins, is involved in coagulation and various metabolic pathways. The enzyme gamma-glutamate carboxylase requires vitamin K to modify MGP to its active carboxylated form [198]. Active MGP inhibits calcification, but the exact mechanism is still quite unclear. The other possible reasons are binding to crystals and blocking more enlargement, or binding to bone morphogenic protein and preventing osteogenic cell differentiation [199]. Higher serum levels of undercarboxylated MGP (ucMGP) are associated with VC, and vitamin K_2_ supplements can increase levels of carboxylated MGP (cMGP) in dialysis patients to reduce VC [104].

### 5.4. Calcimimetics

Calcimimetics are compounds that mimic the effects of calcium and have potential as agents for treating secondary hyperparathyroidism and extraosseous calcifications. The mechanism of calcimimetics in controlling secondary hyperparathyroidism in CKD patients is to bind calcium-sensing receptors (CaRs) in the parathyroid glands, decreasing serum levels of both calcium and phosphate [200]. Calcimimetics are allosteric regulators of CaRs, making the CaRs more sensitive to serum calcium, thereby lowering circulating calcium levels and inhibiting secretion of PTH [201]. From both animal models and human trials, calcimimetics have been demonstrated to reduce VC [201, 202]. In addition, from the ADVANCE study in hemodialysis patients, therapy with a calcimimetic and VDRA was superior in slowing the progression of VC than VDRA therapy alone [202].

### 5.5. Sodium Thiosulfate

Sodium thiosulfate has been used successfully in the treatment of calciphylaxis. It is a small molecule that acts as a vasodilator, antioxidant, and calcium chelator. This compound can reduce inflammation and improve local circulation, to effectively correct VC [203–205]. The mechanism of sodium thiosulfate is to bind to calcium and form calcium thiosulfate, which is highly soluble and can be excreted from the body [203]. In addition, the antioxidant action of sodium thiosulfate may help repair endothelial cell dysfunction and help vasodilatation in injured tissues [206]. A pilot study of sodium thiosulfate in hemodialysis patients for 5 months of treatment indicated that it may be safe to use without apparent side effects [205].

### 5.6. Bisphosphonates

Bisphosphonates have been used successfully to treat osteoporosis for almost 40 years. The mechanism of bisphosphonates in improving bone density is that they, as pyrophosphate analogs, bind to hydroxyapatite and are taken up by osteoclasts then destroy normal osteoclast function to reduce bone resorption [6]. Thus, bisphosphonates should not be used in patients with low bone turnover disorders.

The molecular structure of bisphosphonates, phosphorus-carbon-phosphorus (P-C-P), is very stable because the bonds cannot be hydrolyzed by alkaline phosphatases [207, 208]. The mechanism of bisphosphonates in inhibiting VC remains unclear. They may inhibit osteoclast function, reduce bone resorption, decrease serum calcium and phosphate, and limit their deposition in the vascular wall or their ability to influence the activity of the VSMC NaPi cotransporter [209]. In addition, bisphosphonates can block hydroxyapatite nucleation and growth.

Some case studies indicate that bisphosphonates such as etidronate [210] and pamidronate [208] are useful in treating calciphylaxis (uremic arteriolopathy) in patients with high bone turnover [208], which is a life-threatening complication of CKD. In studies of dialysis patients, etidronate has been found to limit the further progression of VC [207]. However, while alendronate and ibandronate can limit the progression of VC in animal studies, they had no effects in humans [211].

### 5.7. Denosumab

Denosumab is a human IgG2 monoclonal antibody that targets RANKL. Inhibition of RANKL prevents the formation, function, differentiation, and survival of osteoclasts, thereby decreasing bone resorption and bone loss. Thus, denosumab is one of the newest therapies for treating high bone turnover diseases such as osteoporosis [6]. However, it should not be used in patients with low bone turnover. Denosumab reduces calcium release from bone and blocks the direct effects of RANKL on promoting VSMC calcification [157] and TRAP^+^ osteoclast-like cell formation [212]. One study showed that denosumab could reduce VC in a mouse model of glucocorticoid-induced calcification [213].

### 5.8. Teriparatide (Human Parathyroid Hormone 1–34)

Teriparatide is a recombinant form of parathyroid hormone. It is an effective bone formation agent and is used to treat patients with osteoporosis in an intermittent daily administration style [214]. Because PTH can regulate calcium and phosphate levels, it is thought that it might be a way to prevent VC. In addition, teriparatide may increase serum osteopontin levels. In an animal study, teriparatide appeared to reduce the extent of aortic and cardiac valve calcification [94, 214]. Thus, it appeared to be beneficial for both bone and vessels in the absence of hyperparathyroidism.

### 5.9. Avoid Zinc Deficiency

Zinc is a rare element in the human body. Research suggested that high concentrations of phosphate with zinc deficiency could decrease VSMC cell viability, a situation that would increase the risk of VC [215].

### 5.10. RAAS Blocker

Clinical studies have suggested that increased angiotensin II levels in hypertensive patients have a harmful effect by increasing bone resorption and inhibiting mineralization [216]. Angiotensin II downregulates the expression of Cbfa1 but upregulates RANKL expression. By altering the ratio of Cbfa1/RANKL expression via the cAMP-dependent pathway, angiotensin II differently regulates osteoblast and osteoclast differentiation and can lead to enhanced bone resorption and reduced bone formation [217]. Other animal studies showed ARB might exert the potent protective effect on the vascular calcification in CKD as well [218].

## 6. Conclusions

Compared with other populations, CKD patients often have serious and advanced extraosseous calcification, especially in cardiovascular system. Generally, there are two main kinds of VC in CKD: atherosclerosis and arteriosclerosis. However, the pathological processes for atherosclerosis and arteriosclerosis may differ but also overlap. The risk of atherosclerotic events, such as myocardial infarction and stroke, is elevated. In contrast, arteriosclerosis is the predominant pathophysiological process involving fibrosis and thickening of the medial arterial layer, which results in increased arterial stiffness, causing left ventricular hypertrophy, fibrosis, and heart failure. VC is an active and complicated process that may involve numerous mechanisms responsible for leading to osteogenic/chondrogenic conversion of VSMCs in the vascular wall.

Abnormal calcium and phosphate homeostasis is the main reason for stimulating VSMC calcification in patients with CKD. In addition, there are many other factors associated with CKD that may also influence VSMC calcification. These are promoting factors, such as inflammation, calciprotein particles, matrix degradation, and aldosterone, and decreased levels of inhibiting factors such as MGP, fetuin-A, and pyrophosphate that also enhance calcification. In addition, elevated calcium and phosphate can act directly on VSMCs to drive dissimilar and coinciding pathways that predispose to calcification. Recent evidence showed an inverse correlation between VC and bone formation. There is good evidence to suggest that impaired bone turnover, particularly low or high bone turnover, promotes progression of VC. Conversely, VC will enhance the release of sclerostin and sFRP which will attenuate bone turnover and result in increased bone fragility. These hormones also have been identified as possible links between bone and calcification of soft tissues, but a greater understanding of the key elements of VC is still required.

VC can be treated in several ways depending on individual bone turnover status. However, it is necessary to tailor the treatment to each individual. Because the evidence from practice is insufficient, the significance for bone metabolism should be thoughtfully considered in the design of new treatments specifically targeting VC in order to avoid potentially dangerous effects on bone health. Therefore, for CKD patients, retaining benefits for bone health is essential to maintaining good cardiovascular health.

## Figures and Tables

**Table 1:** The possible treatments of vascular calcification in CKD.

General principle	
(1) Treat hypertension, hyperlipidemia, and hyperglycemia as usual	
(2) Body weight control	
(3) Control serum and urine phosphate	
(4) Avoid hypercalcemia	
(5) Avoid magnesium, Iron, and L-lysine deficiency	
(6) Nutritional vitamin-D (NVD) supplement (avoid high dose of VDRAs)	
(7) Possible fractionated heparin for dialysis	
(8) AST-120	

Manipulating the complex biology of vascular calcification	
(1) Pyrophosphate	
(2) Na thiosulfate	
(3) Vit-K (especially in warfarin user)	
(4) Avoid zinc deficiency	
(5) Avoid excess of vit-E, vit-A, vit-C, and fluoride	
(6) Antioxidants (?)	

Patients with high turnover bone disorder (e.g., hyperparathyroid bone disorder; iPTH > 300 pg/mL + high level BAP)	
(1) VDRA (paricalcitol/calcitriol) + NVD (cholecalciferol/ergocalciferol) or calcimimetics + NVD	
(2) Non-metal-containing phosphate binders—sevelamer (for phosphorus)	
(3) Bisphosphonate + VDRA/NVD (for high PTH + hypercalcemia + low bone mass)	
(4) Denosumab + VDRA/NVD (for high PTH + hypercalcemia + low bone mass)	

Patients with low turnover bone disorder (e.g., adynamic bone disorder; bone alkaline phosphatase < 20 ng/mL, and iPTH < 100 pg/mL)	
(1) NVD + low dose VDRA	
(2) Teriparatide	
(3) Non-metal-containing phosphate binders—sevelamer (for oxidative stress and inflammation)	

## References

[1] Matias PJ, Ferreira C, Jorge C (2009). 25-Hydroxyvitamin D3, arterial calcifications and cardiovascular risk markers in haemodialysis patients. *Nephrology Dialysis Transplantation*.

[2] Naves-Díaz M, Álvarez-Hernández D, Passlick-Deetjen J (2008). Oral active vitamin D is associated with improved survival in hemodialysis patients. *Kidney International*.

[3] London GM, Marchais SJ, Guérin AP, Boutouyrie P, Métivier F, De Vernejoul M (2008). Association of bone activity, calcium load, aortic stiffness, and calcifications in ESRD. *Journal of the American Society of Nephrology*.

[4] Rodríguez-García M, Gómez-Alonso C, Naves-Díaz M (2009). Vascular calcifications, vertebral fractures and mortality in haemodialysis patients. *Nephrology Dialysis Transplantation*.

[5] Goldsmith D, Ritz E, Covic A (2004). Vascular calcification: a stiff challenge for the nephrologist—does preventing bone disease cause arterial disease?. *Kidney International*.

[6] Wu M, Rementer C, Giachelli CM (2013). Vascular calcification: an update on mechanisms and challenges in treatment. *Calcified Tissue International*.

[7] Moody WE, Edwards NC, Chue CD, Ferro CJ, Townend JN (2013). Arterial disease in chronic kidney disease. *Heart*.

[8] (2002). K/DOQI clinical practice guidelines for chronic kidney disease: evaluation, classification, and stratification. *The American Journal of Kidney Diseases*.

[9] Eknoyan G, Lameire N, Barsoum R (2004). The burden of kidney disease: improving global outcomes. *Kidney International*.

[10] Mafham M, Emberson J, Landray MJ, Wen C, Baigent C (2011). Estimated glomerular filtration rate and the risk of major vascular events and all-cause mortality: a meta-analysis. *PLoS ONE*.

[11] Kjekshus J, Apetrei E, Barrios V (2007). Rosuvastatin in older patients with systolic heart failure. *The New England Journal of Medicine*.

[12] Shanahan CM, Crouthamel MH, Kapustin A, Giachelli CM (2011). Arterial calcification in chronic kidney disease: key roles for calcium and phosphate. *Circulation Research*.

[13] Frye MA, Melton LJ, Bryant SC (1992). Osteoporosis and calcification of the aorta. *Bone and Mineral*.

[14] Kiel DP, Kauppila LI, Cupples LA, Hannan MT, O'Donnell CJ, Wilson PWF (2001). Bone loss and the progression of abdominal aortic calcification over 25 year period: The Framingham Heart Study. *Calcified Tissue International*.

[15] Vogt MT, San Valentin R, Forrest KY-, Nevitt MC, Cauley JA (1997). Bone mineral density and aortic calcification: the study of osteoporotic fractures. *Journal of the American Geriatrics Society*.

[16] Cannata-Andía JB, Rodríguez-García M, Carrillo-López N, Naves-Díaz M, Díaz-López B (2006). Vascular calcifications: pathogenesis, management, and impact on clinical outcomes. *Journal of the American Society of Nephrology*.

[17] Rodríguez García M, Navez Díaz M, Cannata Andía JB (2005). Bone metabolism, vascular calcifications and mortality: associations beyond mere coincidence. *Journal of Nephrology*.

[18] Cannata-Andia JB, Roman-Garcia P, Hruska K (2011). The connections between vascular calcification and bone health. *Nephrology Dialysis Transplantation*.

[19] Lau WL, Ix JH (2013). Clinical detection, risk factors, and cardiovascular consequences of medial arterial calcification: a pattern of vascular injury associated with aberrant mineral metabolism. *Seminars in Nephrology*.

[20] London GM, Marchais SJ, Guerin AP, Metivier F, Adda H (2002). Arterial structure and function in end-stage renal disease. *Nephrology Dialysis Transplantation*.

[21] Micheletti RG, Fishbein GA, Currier JS, Fishbein MC (2008). Mönckeberg sclerosis revisited: a clarification of the histologic definition of Mönckeberg sclerosis. *Archives of Pathology and Laboratory Medicine*.

[22] Lindbom A (1950). Arteriosclerosis and arterial thrombosis in the lower limb; a roentgenological study. *Acta Radiologica, Supplementum*.

[23] Butany J, Collins MJ, Demellawy DE (2005). Morphological and clinical findings in 247 surgically excised native aortic valves. *The Canadian Journal of Cardiology*.

[24] Otto CM, Kuusisto J, Reichenbach DD, Gown AM, O'Brien KD (1994). Characterization of the early lesion of “degenerative” valvular aortic stenosis: histological and immunohistochemical studies. *Circulation*.

[25] Wallby L, Janerot-Sjöberg B, Steffensen T, Broqvist M (2002). T lymphocyte infiltration in non-rheumatic aortic stenosis: a comparative descriptive study between tricuspid and bicuspid aortic valves. *Heart*.

[26] Rajamannan NM, Subramaniam M, Rickard D (2003). Human aortic valve calcification is associated with an osteoblast phenotype. *Circulation*.

[27] Mohler ER, Chawla MK, Chang AW (1999). Identification and characterization of calcifying valve cells from human and canine aortic valves. *Journal of Heart Valve Disease*.

[28] Mohler ER, Gannon F, Reynolds C, Zimmerman R, Keane MG, Kaplan FS (2001). Bone formation and inflammation in cardiac valves. *Circulation*.

[29] Leopold JA (2012). Cellular mechanisms of aortic valve calcification. *Circulation: Cardiovascular Interventions*.

[30] Towler DA (2013). Molecular and cellular aspects of calcific aortic valve disease. *Circulation Research*.

[31] Rajamannan NM, Evans FJ, Aikawa E (2011). Calcific aortic valve disease: not simply a degenerative process: a review and agenda for research from the national heart and lung and blood institute aortic stenosis working group. *Circulation*.

[32] Karwowski W, Naumnik B, Szczepański M, Myśliwiec M (2012). The mechanism of vascular calcification—a systematic review. *Medical Science Monitor*.

[33] Ix JH, Chertow GM, Shlipak MG, Brandenburg VM, Ketteler M, Whooley MA (2007). Association of fetuin-A with mitral annular calcification and aortic stenosis among persons with coronary heart disease: data from the heart and soul study. *Circulation*.

[34] Ott SM (2013). Therapy for patients with CKD and low bone mineral density. *Nature Reviews. Nephrology*.

[35] Mathew S, Tustison KS, Sugatani T, Chaudhary LR, Rifas L, Hruska KA (2008). The mechanism of phosphorus as a cardiovascular risk factor in CKD. *Journal of the American Society of Nephrology*.

[36] Reynolds JL, Joannides AJ, Skepper JN (2004). Human vascular smooth muscle cells undergo vesicle-mediated calcification in response to changes in extracellular calcium and phosphate concentrations: a potential mechanism for accelerated vascular calcification in ESRD. *Journal of the American Society of Nephrology*.

[37] Schoppet M, Shroff RC, Hofbauer LC, Shanahan CM (2008). Exploring the biology of vascular calcification in chronic kidney disease: what's circulating?. *Kidney International*.

[38] Hruska KA, Mathew S, Lund R, Qiu P, Pratt R (2008). Hyperphosphatemia of chronic kidney disease. *Kidney International*.

[39] Marks J, Debnam ES, Unwin RJ (2010). Phosphate homeostasis and the renal-gastrointestinal axis. *American Journal of Physiology—Renal Physiology*.

[40] Gattineni J, Bates C, Twombley K (2009). FGF23 decreases renal NaPi-2a and NaPi-2c expression and induces hypophosphatemia in vivo predominantly via FGF receptor 1. *The American Journal of Physiology—Renal Physiology*.

[41] Segawa H, Yamanaka S, Ito M (2005). Internalization of renal type IIc Na-Pi cotransporter in response to a high-phosphate diet. *The American Journal of Physiology—Renal Physiology*.

[42] Segawa H, Yamanaka S, Onitsuka A (2007). Parathyroid hormone-dependent endocytosis of renal type IIc Na-P_i_ cotransporter. *American Journal of Physiology: Renal Physiology*.

[43] Hu MC, Shi M, Zhang J (2010). Klotho: A novel phosphaturic substance acting as an autocrine enzyme in the renal proximal tubule. *The FASEB Journal*.

[44] Rucker RB (1974). Calcium binding to elastin. *Advances in Experimental Medicine and Biology*.

[45] Figueiredo CP, Rajamannan NM, Lopes JB (2013). Serum phosphate and hip bone mineral density as additional factors for high vascular calcification scores in a community-dwelling: the São Paulo Ageing & Health Study (SPAH). *Bone*.

[46] Román-García P, Carrillo-López N, Fernández-Martín JL, Naves-Díaz M, Ruiz-Torres MP, Cannata-Andía JB (2010). High phosphorus diet induces vascular calcification, a related decrease in bone mass and changes in the aortic gene expression. *Bone*.

[47] Ix JH, Katz R, Kestenbaum BR (2012). Fibroblast growth factor-23 and death, heart failure, and cardiovascular events in community-living individuals: CHS (Cardiovascular Health Study). *Journal of the American College of Cardiology*.

[48] Isakova T, Wahl P, Vargas GS (2011). Fibroblast growth factor 23 is elevated before parathyroid hormone and phosphate in chronic kidney disease. *Kidney International*.

[49] Perwad F, Zhang MY, Tenenhouse HS, Portale AA (2007). Fibroblast growth factor 23 impairs phosphorus and vitamin D metabolism in vivo and suppresses 25-hydroxyvitamin D-1*α*-hydroxylase expression in vitro. *American Journal of Physiology: Renal Physiology*.

[50] Wesseling-Perry K, Jüppner H (2013). The osteocyte in CKD: new concepts regarding the role of FGF23 in mineral metabolism and systemic complications. *Bone*.

[51] House SJ, Potier M, Bisaillon J, Singer HA, Trebak M (2008). The non-excitable smooth muscle: calcium signaling and phenotypic switching during vascular disease. *Pflugers Archiv European Journal of Physiology*.

[52] Sammels E, Parys JB, Missiaen L, de Smedt H, Bultynck G (2010). Intracellular Ca2+ storage in health and disease: a dynamic equilibrium. *Cell Calcium*.

[53] Shanahan CM, Weissberg PL, Metcalfe JC (1993). Isolation of gene markers of differentiated and proliferating vascular smooth muscle cells. *Circulation Research*.

[54] Burger D, Levin A (2013). “Shedding” light on mechanisms of hyperphosphatemic vascular dysfunction. *Kidney International*.

[55] Jono S, McKee CE, Shioi A (2000). Phosphate regulation of vascular smooth muscle cell calcification. *Circulation Research*.

[56] Steitz SA, Speer MY, Curinga G (2001). Smooth muscle cell phenotypic transition associated with calcification: upregulation of Cbfa1 and downregulation of smooth muscle lineage markers. *Circulation Research*.

[57] Wada T, McKee S, Giachelli CM, McKee MD, Steitz S (1999). Calcification of vascular smooth muscle cell cultures: inhibition by osteopontin. *Circulation Research*.

[58] Takei Y, Yamamoto H, Sato T (2012). Stanniocalcin 2 is associated with ectopic calcification in *α*-klotho mutant mice and inhibits hyperphosphatemia-induced calcification in aortic vascular smooth muscle cells. *Bone*.

[59] Martinez-Moreno JM, Munoz-Castaneda JR, Herencia C (2012). In vascular smooth muscle cells paricalcitol prevents phosphate-induced Wnt/*β*-catenin activation. *American Journal of Physiology: Renal Physiology*.

[60] Mikhaylova L, Malmquist J, Nurminskaya M (2007). Regulation of in vitro vascular calcification by BMP4, VEGF and Wnt3a. *Calcified Tissue International*.

[61] Kamiya N, Kobayashi T, Mochida Y (2010). Wnt inhibitors Dkk1 and Sost are downstream targets of BMP signaling through the type IA receptor (BMPRIA) in osteoblasts. *Journal of Bone and Mineral Research*.

[62] Shroff RC, McNair R, Skepper JN (2010). Chronic mineral dysregulation promotes vascular smooth muscle cell adaptation and extracellular matrix calcification. *Journal of the American Society of Nephrology*.

[63] Schlieper G, Aretz A, Verberckmoes SC (2010). Ultrastructural analysis of vascular calcifications in uremia. *Journal of the American Society of Nephrology*.

[64] Heiss A, Eckert T, Aretz A (2008). Hierarchical role of fetuin-A and acidic serum proteins in the formation and stabilization of calcium phosphate particles. *The Journal of Biological Chemistry*.

[65] Hamano T, Matsui I, Mikami S (2010). Fetuin-mineral complex reflects extraosseous calcification stress in CKD. *Journal of the American Society of Nephrology*.

[66] El-Abbadi MM, Pai AS, Leaf EM (2009). Phosphate feeding induces arterial medial calcification in uremic mice: role of serum phosphorus, fibroblast growth factor-23, and osteopontin. *Kidney International*.

[67] Shanahan CM, Cary NR, Salisbury JR, Proudfoot D, Weissberg PL, Edmonds ME (1999). Medial localization of mineralization-regulating proteins in association with Monckeberg's sclerosis: evidence for smooth muscle cell-mediated vascular calcification. *Circulation*.

[68] Pai A, Leaf EM, El-Abbadi M, Giachelli CM (2011). Elastin degradation and vascular smooth muscle cell phenotype change precede cell loss and arterial medial calcification in a uremic mouse model of chronic kidney disease. *The American Journal of Pathology*.

[69] Simionescu A, Philips K, Vyavahare N (2005). Elastin-derived peptides and TGF-beta1 induce osteogenic responses in smooth muscle cells. *Biochemical and Biophysical Research Communications*.

[70] Heldin CH, Miyazono K, ten Dijke P (1997). TGF-beta signalling from cell membrane to nucleus through SMAD proteins. *Nature*.

[71] Lee KS, Kim HJ, Li QL (2000). Runx2 is a common target of transforming growth factor beta1 and bone morphogenetic protein 2, and cooperation between Runx2 and Smad5 induces osteoblast-specific gene expression in the pluripotent mesenchymal precursor cell line C2C12. *Molecular and Cellular Biology*.

[72] Shanahan CM, Cary NR, Metcalfe JC, Weissberg PL (1994). High expression of genes for calcification-regulating proteins in human atherosclerotic plaques. *The Journal of Clinical Investigation*.

[73] Spronk HM, Soute BA, Schurgers LJ (2001). Matrix Gla protein accumulates at the border of regions of calcification and normal tissue in the media of the arterial vessel wall. *Biochemical and Biophysical Research Communications*.

[74] Lomashvili KA, Wang X, Wallin R, O'Neill WC (2011). Matrix Gla protein metabolism in vascular smooth muscle and role in uremic vascular calcification. *The Journal of Biological Chemistry*.

[75] Murshed M, Schinke T, McKee G, McKee MD, Karsenty G (2004). Extracellular matrix mineralization is regulated locally; different roles of two gla-containing proteins. *The Journal of Cell Biology*.

[76] Leonard O, Spaak J, Goldsmith D (2013). Regression of vascular calcification in chronic kidney disease—feasible or fantasy? A review of the clinical evidence. *British Journal of Clinical Pharmacology*.

[77] Luo G, Ducy P, McKee MD (1997). Spontaneous calcification of arteries and cartilage in mice lacking matrix GLA protein. *Nature*.

[78] Schinke T, Amendt C, Trindl A, Poschke O, Muller-Esterl W, Jahnen-Dechent W (1996). The serum protein alpha2-HS glycoprotein/fetuin inhibits apatite formation in vitro and in mineralizing calvaria cells. A possible role in mineralization and calcium homeostasis. *The Journal of Biological Chemistry*.

[79] Heiss A, DuChesne A, Denecke B (2003). Structural basis of calcification inhibition by alpha 2-HS glycoprotein/fetuin-A. Formation of colloidal calciprotein particles. *The Journal of Biological Chemistry*.

[80] Reynolds JL, Skepper JN, McNair R (2005). Multifunctional roles for serum protein fetuin-a in inhibition of human vascular smooth muscle cell calcification. *Journal of the American Society of Nephrology*.

[81] Moe SM, Reslerova M, Ketteler M (2005). Role of calcification inhibitors in the pathogenesis of vascular calcification in chronic kidney disease (CKD). *Kidney International*.

[82] Ketteler M, Bongartz P, Westenfeld R (2003). Association of low fetuin-A (AHSG) concentrations in serum with cardiovascular mortality in patients on dialysis: A Cross-Sectional Study. *The Lancet*.

[83] Odamaki M, Shibata T, Takita T, Kumagai H (2005). Serum fetuin-A and aortic calcification in hemodialysis patients. *Kidney International*.

[84] Stenvinkel P, Wang K, Qureshi AR (2005). Low fetuin-A levels are associated with cardiovascular death: impact of variations in the gene encoding fetuin. *Kidney International*.

[85] Lomashvili KA, Khawandi W, O’Neill WC (2005). Reduced plasma pyrophosphate levels in hemodialysis patients. *Journal of the American Society of Nephrology*.

[86] O'Neill WC, Sigrist MK, McIntyre CW (2010). Plasma pyrophosphate and vascular calcification in chronic kidney disease. *Nephrology, Dialysis, Transplantation*.

[87] Tintut Y, Patel J, Parhami F, Demer LL (2000). Tumor necrosis factor-alpha promotes in vitro calcification of vascular cells via the cAMP pathway. *Circulation*.

[88] Al-Aly Z, Shao JS, Lai CF (2007). Aortic Msx2-Wnt calcification cascade is regulated by TNF-alpha-dependent signals in diabetic Ldlr-/- mice. *Arteriosclerosis, Thrombosis, and Vascular Biology*.

[89] Towler DA, Shao JS, Cheng SL, Pingsterhaus JM, Loewy AP (2006). Osteogenic regulation of vascular calcification. *Annals of the New York Academy of Sciences*.

[90] Lacativa PG, Farias ML (2010). Osteoporosis and inflammation. *Arquivos Brasileiros de Endocrinologia e Metabologia*.

[91] Byon CH, Javed A, Dai Q (2008). Oxidative stress induces vascular calcification through modulation of the osteogenic transcription factor Runx2 by AKT signaling. *The Journal of Biological Chemistry*.

[92] Zebboudj AF, Shin V, Bostrom K (2003). Matrix GLA protein and BMP-2 regulate osteoinduction in calcifying vascular cells. *Journal of Cellular Biochemistry*.

[93] Jono S, Nishizawa Y, Shioi A, Morii H (1998). 1,25-Dihydroxyvitamin D3 increases in vitro vascular calcification by modulating secretion of endogenous parathyroid hormone-related peptide. *Circulation*.

[94] Shao JS, Cheng SL, Charlton-Kachigian N, Loewy AP, Towler DA (2003). Teriparatide (human parathyroid hormone (1-34)) inhibits osteogenic vascular calcification in diabetic low density lipoprotein receptor-deficient mice. *The Journal of Biological Chemistry*.

[95] Vattikuti R, Towler DA (2004). Osteogenic regulation of vascular calcification: an early perspective. *American Journal of Physiology: Endocrinology and Metabolism*.

[96] Torres PA, De Broe M (2012). Calcium-sensing receptor, calcimimetics, and cardiovascular calcifications in chronic kidney disease. *Kidney International*.

[97] Johnson RC, Leopold JA, Loscalzo J (2006). Vascular calcification: pathobiological mechanisms and clinical implications. *Circulation Research*.

[98] Coen G, Ballanti P, Mantella D (2009). Bone turnover, osteopenia and vascular calcifications in hemodialysis patients. A histomorphometric and multislice CT study. *The American Journal of Nephrology*.

[99] Graciolli FG, Neves KR, dos Reis LM (2009). Phosphorus overload and PTH induce aortic expression of Runx2 in experimental uraemia. *Nephrology, Dialysis, Transplantation*.

[100] Salem S, Bruck H, Bahlmann FH (2012). Relationship between magnesium and clinical biomarkers on inhibition of vascular calcification. *The American Journal of Nephrology*.

[101] Ketteler M, Rothe H, Krüger T, Biggar PH, Schlieper G (2011). Mechanisms and treatment of extraosseous calcification in chronic kidney disease. *Nature Reviews Nephrology*.

[102] Krueger T, Westenfeld R, Ketteler M, Schurgers LJ, Floege J (2009). Vitamin K deficiency in CKD patients: a modifiable risk factor for vascular calcification?. *Kidney International*.

[103] Nakagawa K, Hirota Y, Sawada N (2010). Identification of UBIAD1 as a novel human menaquinone-4 biosynthetic enzyme. *Nature*.

[104] Schlieper G, Westenfeld R, Krüger T (2011). Circulating nonphosphorylated carboxylated matrix Gla protein predicts survival in ESRD. *Journal of the American Society of Nephrology*.

[105] Alonso A, Lopez FL, Matsushita K (2011). Chronic kidney disease is associated with the incidence of atrial fibrillation: the atherosclerosis risk in communities (ARIC) study. *Circulation*.

[106] Price PA, Faus SA, Williamson MK (1998). Warfarin causes rapid calcification of the elastic lamellae in rat arteries and heart valves. *Arteriosclerosis, Thrombosis, and Vascular Biology*.

[107] Coates T, Kirkland GS, Dymock RB (1998). Cutaneous necrosis from calcific uremic arteriolopathy. *American Journal of Kidney Diseases*.

[108] Hayashi M, Takamatsu I, Kanno Y, Yoshida T, Abe T, Sato Y (2012). A case-control study of calciphylaxis in Japanese end-stage renal disease patients. *Nephrology Dialysis Transplantation*.

[109] Meng Y, Zhang H, Li Y, Li Q, Zuo L (2014). Effects of unfractionated heparin on renal osteodystrophy and vascular calcification in chronic kidney disease rats. *Bone*.

[110] Green DE (1933). The potentials of ascorbic acid. *The Biochemical Journal*.

[111] Franceschi RT (1992). The role of ascorbic acid in mesenchymal differentiation. *Nutrition Reviews*.

[112] Altaf FM, Hering TM, Kazmi NH, Yoo JU, Johnstone B (2006). Ascorbate-enhanced chondrogenesis of ATDC5 cells. *European Cells and Materials*.

[113] Farquharson C, Berry JL, Mawer EB, Seawright E, Whitehead CC (1998). Ascorbic acid-induced chondrocyte terminal differentiation: the role of the extracellular matrix and 1,25-dihydroxyvitamin D. *European Journal of Cell Biology*.

[114] Ciceri P, Volpi E, Brenna I (2012). Combined effects of ascorbic acid and phosphate on rat VSMC osteoblastic differentiation. *Nephrology Dialysis Transplantation*.

[115] Roodman GD (2012). Vitamin E: good for the heart, bad for the bones?. *Nature Medicine*.

[116] Fujita K, Iwasaki M, Ochi H (2012). Vitamin E decreases bone mass by stimulating osteoclast fusion. *Nature Medicine*.

[117] Kidney Disease: Improving Global Outcomes (KDIGO) CKD-MBD Work Group (2009). KDIGO clinical practice guideline for the diagnosis, evaluation, prevention, and treatment of Chronic Kidney Disease-Mineral and Bone Disorder (CKD-MBD). *Kidney International. Supplement*.

[118] Price PA, Omid N, Than TN, Williamson MK (2002). The amino bisphosphonate ibandronate prevents calciphylaxis in the rat at doses that inhibit bone resorption. *Calcified Tissue International*.

[119] Haffner D, Hocher B, Müller D (2005). Systemic cardiovascular disease in uremic rats induced by 1,25(OH) 2D3. *Journal of Hypertension*.

[120] Mizobuchi M, Ogata H, Koiwa F, Kinugasa E, Akizawa T (2009). Vitamin D and vascular calcification in chronic kidney disease. *Bone*.

[121] Mizobuchi M, Finch JL, Martin DR, Slatopolsky E (2007). Differential effects of vitamin D receptor activators on vascular calcification in uremic rats. *Kidney International*.

[122] Suda T, Takahashi F, Takahashi N (2012). Bone effects of vitamin D—discrepancies between in vivo and in vitro studies. *Archives of Biochemistry and Biophysics*.

[123] Lomashvili KA, Wang X, O'Neill WC (2014). Role of local versus systemic vitamin D receptors in vascular calcification. *Arteriosclerosis, Thrombosis, and Vascular Biology*.

[124] Inoue T, Kawashima H (1988). 1,25-dihydroxyvitamin D_3_ stimulates ^45^Ca2^+^-uptake by cultured vascular smooth muscle cells derived from rat aorta. *Biochemical and Biophysical Research Communications*.

[125] Mathew S, Lund RJ, Chaudhary LR, Geurs T, Hruska KA (2008). Vitamin D receptor activators can protect against vascular calcification. *Journal of the American Society of Nephrology*.

[126] Shroff R, Egerton M, Bridel M (2008). A bimodal association of vitamin d levels and vascular disease in children on dialysis. *Journal of the American Society of Nephrology*.

[127] Levin A, Yan CL (2005). Vitamin D and its analogues: do they protect against cardiovascular disease in patients with kidney disease?. *Kidney International*.

[128] Kovesdy CP, Ahmadzadeh S, Anderson JE, Kalantar-Zadeh K (2008). Association of activated vitamin D treatment and mortality in chronic kidney disease. *Archives of Internal Medicine*.

[129] Quarles LD (2013). A systems biology preview of the relationships between mineral and metabolic complications in chronic kidney disease. *Seminars in Nephrology*.

[130] Wu SY, Yu YR, Cai Y (2012). Endogenous aldosterone is involved in vascular calcification in rat. *Experimental Biology and Medicine (Maywood)*.

[131] Jaffe IZ, Tintut Y, Newfell BG, Demer LL, Mendelsohn ME (2007). Mineralocorticoid receptor activation promotes vascular cell calcification. *Arteriosclerosis, Thrombosis, and Vascular Biology*.

[132] Cooper JN, Tepper P, Barinas-Mitchell E, Woodard GA, Sutton-Tyrrell K (2012). Serum aldosterone is associated with inflammation and aortic stiffness in normotensive overweight and obese young adults. *Clinical and Experimental Hypertension*.

[133] de Rita O, Hackam DG, Spence JD (2012). Effects of aldosterone on human atherosclerosis: plasma aldosterone and progression of carotid plaque. *Canadian Journal of Cardiology*.

[134] Hillaert MA, Lentjes EG, Kemperman H (2013). Aldosterone, atherosclerosis and vascular events in patients with stable coronary artery disease. *International Journal of Cardiology*.

[135] Lim K, Lu T, Molostvov G (2012). Vascular klotho deficiency potentiates the development of human artery calcification and mediates resistance to fibroblast growth factor 23. *Circulation*.

[136] Sanz-Rosa D, Cediel E, de Las Heras N (2005). Participation of aldosterone in the vascular inflammatory response of spontaneously hypertensive rats: role of the NF*κ*B/I*κ*B system. *Journal of Hypertension*.

[137] Al-Aly Z (2008). Vascular calcification in uremia: what is new and where are we going?. *Advances in Chronic Kidney Disease*.

[138] Lee H, Woo KM, Ryoo H, Baek J (2010). Tumor necrosis factor-*α* increases alkaline phosphatase expression in vascular smooth muscle cells via MSX2 induction. *Biochemical and Biophysical Research Communications*.

[139] Sun Y, Byon CH, Yuan K (2012). Smooth muscle cell-specific runx2 deficiency inhibits vascular calcification. *Circulation Research*.

[140] Chen K, Rajewsky N (2007). The evolution of gene regulation by transcription factors and microRNAs. *Nature Reviews Genetics*.

[141] Bartel DP (2009). MicroRNAs: target recognition and regulatory functions. *Cell*.

[142] Small EM, Olson EN (2011). Pervasive roles of microRNAs in cardiovascular biology. *Nature*.

[143] Taibi F, Metzinger-Le Meuth V, M'Baya-Moutoula E (2014). Possible involvement of microRNAs in vascular damage in experimental chronic kidney disease. *Biochimica et Biophysica Acta*.

[144] Chen NX, Kiattisunthorn K, O'Neill KD (2013). Decreased microRNA is involved in the vascular remodeling abnormalities in chronic kidney disease (CKD). *PLoS ONE*.

[145] Iwasaki Y, Yamato H, Nii-Kono T (2006). Administration of oral charcoal adsorbent (AST-120) suppresses low-turnover bone progression in uraemic rats. *Nephrology Dialysis Transplantation*.

[146] Barreto FC, Barreto DV, Liabeuf S (2009). Serum indoxyl sulfate is associated with vascular disease and mortality in chronic kidney disease patients. *Clinical Journal of the American Society of Nephrology*.

[147] Mozar A, Louvet L, Godin C (2012). Indoxyl sulphate inhibits osteoclast differentiation and function. *Nephrology Dialysis Transplantation*.

[148] Kim YH, Kwak KA, Gil HW, Song HY, Hong SY (2013). Indoxyl sulfate promotes apoptosis in cultured osteoblast cells. *BMC Pharmacology & Toxicology*.

[149] Nii-Kono T, Iwasaki Y, Uchida M (2007). Indoxyl sulfate induces skeletal resistance to parathyroid hormone in cultured osteoblastic cells. *Kidney International*.

[150] Iwasaki Y, Kazama JJ, Yamato H, Shimoda H, Fukagawa M (2013). Accumulated uremic toxins attenuate bone mechanical properties in rats with chronic kidney disease. *Bone*.

[151] Goto S, Kitamura K, Kono K, Nakai K, Fujii H, Nishi S (2013). Association between AST-120 and abdominal aortic calcification in predialysis patients with chronic kidney disease. *Clinical and Experimental Nephrology*.

[152] Boström KI, Rajamannan NM, Towler DA (2011). The regulation of valvular and vascular sclerosis by osteogenic morphogens. *Circulation Research*.

[153] Liu C, Walter TS, Huang P (2010). Structural and functional insights of RANKL-RANK interaction and signaling. *The Journal of Immunology*.

[154] Heymann M, Herisson F, Davaine J (2012). Role of the OPG/RANK/RANKL triad in calcifications of the atheromatous plaques: comparison between carotid and femoral beds. *Cytokine*.

[155] Steinmetz M, Skowasch D, Wernert N (2008). Differential profile of the OPG/RANKL/RANK-system in degenerative aortic native and bioprosthetic valves. *The Journal of Heart Valve Disease*.

[156] Boyce BF, Xing L (2007). Biology of RANK, RANKL, and osteoprotegerin. *Arthritis Research and Therapy*.

[157] Panizo S, Cardus A, Encinas M (2009). RANKL increases vascular smooth muscle cell calcification through a rank-bmp4-dependent pathway. *Circulation Research*.

[158] Shargorodsky M, Boaz M, Luckish A, Matas Z, Gavish D, Mashavi M (2009). Osteoprotegerin as an independent marker of subclinical atherosclerosis in osteoporotic postmenopausal women. *Atherosclerosis*.

[159] Demer LL, Tintut Y (2008). Vascular calcification: pathobiology of a multifaceted disease. *Circulation*.

[160] Mathew S, Davies M, Lund R, Saab G, Hruska KA (2006). Function and effect of bone morphogenetic protein-7 in kidney bone and the bone-vascular links in chronic kidney disease. *European Journal of Clinical Investigation*.

[161] Barreto DV, Barreto FDC, de Carvalho AB (2008). Association of changes in bone remodeling and coronary calcification in hemodialysis patients: a prospective study. *The American Journal of Kidney Diseases*.

[162] Adragao T, Herberth J, Monier-Faugere M (2009). Low bone volume—a risk factor for coronary calcifications in hemodialysis patients. *Clinical Journal of the American Society of Nephrology*.

[163] Moe S, Drüeke T, Cunningham J (2006). Definition, evaluation, and classification of renal osteodystrophy: a position statement from Kidney Disease: improving Global Outcomes (KDIGO). *Kidney International*.

[164] Silverman SL (2010). Sclerostin. *Journal of Osteoporosis*.

[165] Baron R, Rawadi G (2007). Targeting the Wnt/*β*-catenin pathway to regulate bone formation in the adult skeleton. *Endocrinology*.

[166] Wei W, Zeve D, Suh JM (2011). Biphasic and dosage-dependent regulation of osteoclastogenesis by *β*-catenin. *Molecular and Cellular Biology*.

[167] Viaene L, Behets GJ, Claes K (2013). Sclerostin: another bone-related protein related to all-cause mortality in haemodialysis?. *Nephrology, Dialysis, Transplantation*.

[168] Surendran K, Schiavi S, Hruska KA (2005). Wnt-dependent *β*-catenin signaling is activated after unilateral ureteral obstruction, and recombinant secreted frizzled-related protein 4 alters the progression of renal fibrosis. *Journal of the American Society of Nephrology*.

[169] Hampson G, Edwards S, Conroy S, Blake GM, Fogelman I, Frost ML (2013). The relationship between inhibitors of the Wnt signalling pathway (Dickkopf-1(DKK1) and sclerostin), bone mineral density, vascular calcification and arterial stiffness in post-menopausal women. *Bone*.

[170] Pinzone JJ, Hall BM, Thudi NK (2009). The role of Dickkopf-1 in bone development, homeostasis, and disease. *Blood*.

[171] Poole KES, van Bezooijen RL, Loveridge N (2005). Sclerostin is a delayed secreted product of osteocytes that inhibits bone formation. *FASEB Journal*.

[172] Cejka D, Herberth J, Branscum AJ (2011). Sclerostin and dickkopf-1 in renal osteodystrophy. *Clinical Journal of the American Society of Nephrology*.

[173] Caira FC, Stock SR, Gleason TG (2006). Human degenerative valve disease is associated with up-regulation of low-density lipoprotein receptor-related protein 5 receptor-mediated bone formation. *Journal of the American College of Cardiology*.

[174] Shao J, Cheng S, Pingsterhaus JM, Charlton-Kachigian N, Loewy AP, Towler DA (2005). Msx2 promotes cardiovascular calcification by activating paracrine Wnt signals. *Journal of Clinical Investigation*.

[175] Zhu D, Mackenzie NCW, Millán JL, Farquharson C, MacRae VE (2011). The appearance and modulation of osteocyte marker expression during calcification of vascular smooth muscle cells. *PLoS ONE*.

[176] Drake MT, Srinivasan B, Mödder UI (2010). Effects of parathyroid hormone treatment on circulating sclerostin levels in postmenopausal women. *The Journal of Clinical Endocrinology & Metabolism*.

[177] Drüeke TB, Lafage-Proust MH (2011). Sclerostin: just one more player in renal bone disease?. *Clinical Journal of the American Society of Nephrology*.

[178] Mödder UI, Hoey KA, Amin S (2011). Relation of age, gender, and bone mass to circulating sclerostin levels in women and men. *Journal of Bone and Mineral Research*.

[179] Ardawi M-SM, Al-Kadi HA, Rouzi AA, Qari MH (2011). Determinants of serum sclerostin in healthy pre- and postmenopausal women. *Journal of Bone and Mineral Research*.

[180] García-Martín A, Rozas-Moreno P, Reyes-García R (2012). Circulating levels of sclerostin are increased in patients with type 2 diabetes mellitus. *The Journal of Clinical Endocrinology and Metabolism*.

[181] Schulz E, Arfai K, Liu X, Sayre J, Gilsanz V (2004). Aortic calcification and the risk of osteoporosis and fractures. *The Journal of Clinical Endocrinology and Metabolism*.

[182] Atkins GJ, Rowe PS, Lim HP (2011). Sclerostin is a locally acting regulator of late-osteoblast/preosteocyte differentiation and regulates mineralization through a MEPE-ASARM-dependent mechanism. *Journal of Bone and Mineral Research*.

[183] Malberti F (2013). Hyperphosphataemia: treatment options. *Drugs*.

[184] de Oliveira RB, Graciolli FG, dos Reis LM (2013). Disturbances of Wnt/*β*-catenin pathway and energy metabolism in early CKD: effect of phosphate binders. *Nephrology, Dialysis, Transplantation*.

[185] Hutchison AJ, Smith CP, Brenchley PEC (2011). Pharmacology, efficacy and safety of oral phosphate binders. *Nature Reviews Nephrology*.

[186] Qunibi W, Moustafa M, Muenz LR (2008). A 1-year randomized trial of calcium acetate versus sevelamer on progression of coronary artery calcification in hemodialysis patients with comparable lipid control: the Calcium Acetate Renagel Evaluation-2 (CARE-2) study. *American Journal of Kidney Diseases*.

[187] Phan O, Maillard M, Peregaux C (2013). PA21, a new iron-based noncalcium phosphate binder, prevents vascular calcification in chronic renal failure rats. *The Journal of Pharmacology and Experimental Therapeutics*.

[188] Striker GE (2009). Beyond phosphate binding: the effect of binder therapy on novel biomarkers may have clinical implications for the management of chronic kidney disease patients. *Kidney International*.

[189] Wolf M, Shah A, Gutierrez O (2007). Vitamin D levels and early mortality among incident hemodialysis patients. *Kidney International*.

[190] Lau WL, Leaf EM, Hu MC (2012). Vitamin D receptor agonists increase klotho and osteopontin while decreasing aortic calcification in mice with chronic kidney disease fed a high phosphate diet. *Kidney International*.

[191] Teng M, Wolf M, Ofsthun MN (2005). Activated injectable vitamin D and hemodialysis survival: a historical cohort study. *Journal of the American Society of Nephrology*.

[192] Teng M, Wolf M, Lowrie E, Ofsthun N, Lazarus JM, Thadhani R (2003). Survival of patients undergoing hemodialysis with paricalcitol or calcitriol therapy. *The New England Journal of Medicine*.

[193] Ryan JW, Anderson PH, Turner AG, Morris HA (2013). Vitamin D activities and metabolic bone disease. *Clinica Chimica Acta*.

[194] Anderson PH, Lam NN, Turner AG (2013). The pleiotropic effects of vitamin D in bone. *The Journal of Steroid Biochemistry and Molecular Biology*.

[195] Lieben L, Carmeliet G, Masuyama R (2011). Calcemic actions of vitamin D: effects on the intestine, kidney and bone. *Best Practice & Research Clinical Endocrinology & Metabolism*.

[196] Bosworth C, De Boer IH (2013). Impaired vitamin D metabolism in CKD. *Seminars in Nephrology*.

[197] Bell NH, Shaw S, Turner RT (1984). Evidence that 1,25-dihydroxyvitamin D3 inhibits the hepatic production of 25-hydroxyvitamin D in man. *The Journal of Clinical Investigation*.

[198] Westenfeld R, Krueger T, Schlieper G (2012). Effect of vitamin K_2_ supplementation on functional vitamin K deficiency in hemodialysis patients: a randomized trial. *American Journal of Kidney Diseases*.

[199] Spronk HM, Soute BA, Schurgers LJ, Thijssen HH, De Mey JG, Vermeer C (2003). Tissue-specific utilization of menaquinone-4 results in the prevention of arterial calcification in warfarin-treated rats. *Journal of Vascular Research*.

[200] Block GA, Martin KJ, de Francisco ALM (2004). Cinacalcet for secondary hyperparathyroidism in patients receiving hemodialysis. *The New England Journal of Medicine*.

[201] Jung S, Querfeld U, Müller D, Rudolph B, Peters H, Krämer S (2012). Submaximal suppression of parathyroid hormone ameliorates calcitriol-induced aortic calcification and remodeling and myocardial fibrosis in uremic rats. *Journal of Hypertension*.

[202] Raggi P, Chertow GM, Torres PU (2011). The ADVANCE study: a randomized study to evaluate the effects of cinacalcet plus low-dose vitamin D on vascular calcification in patients on hemodialysis. *Nephrology, Dialysis, Transplantation*.

[203] Ong S, Coulson IH (2012). Diagnosis and treatment of calciphylaxis. *Skinmed*.

[204] Pasch A, Schaffner T, Huynh-Do U, Frey BM, Frey FJ, Farese S (2008). Sodium thiosulfate prevents vascular calcifications in uremic rats. *Kidney International*.

[205] Mathews SJ, de Las Fuentes L, Podaralla P (2011). Effects of sodium thiosulfate on vascular calcification in end-stage renal disease: a pilot study of feasibility, safety and efficacy. *American Journal of Nephrology*.

[206] Schlieper G, Brandenburg V, Ketteler M, Floege J (2009). Sodium thiosulfate in the treatment of calcific uremic arteriolopathy. *Nature Reviews Nephrology*.

[207] Nitta K, Akiba T, Suzuki K (2004). Effects of cyclic intermittent etidronate therapy on coronary artery calcification in patients receiving long-term hemodialysis. *The American Journal of Kidney Diseases*.

[208] Monney P, Nguyen QV, Perroud H, Descombes E (2004). Rapid improvement of calciphylaxis after intravenous pamidronate therapy in a patient with chronic renal failure. *Nephrology, Dialysis, Transplantation*.

[209] Liu WC, Yen JF, Lang CL, Yan MT, Lu KC (2013). Bisphophonates in CKD patients with low bone mineral density. *The Scientific World Journal*.

[210] Shiraishi N, Kitamura K, Miyoshi T (2006). Successful treatment of a patient with severe calcific uremic arteriolopathy (calciphylaxis) by etidronate disodium. *The American Journal of Kidney Diseases*.

[211] Toussaint ND, Lau KK, Strauss BJ, Polkinghorne KR, Kerr PG (2010). Effect of alendronate on vascular calcification in CKD stages 3 and 4: a pilot randomized controlled trial. *American Journal of Kidney Diseases*.

[212] Byon CH, Sun Y, Chen J (2011). Runx2-upregulated receptor activator of nuclear factor kappaB ligand in calcifying smooth muscle cells promotes migration and osteoclastic differentiation of macrophages. *Arteriosclerosis, Thrombosis and Vascular Biology*.

[213] Helas S, Goettsch C, Schoppet M (2009). Inhibition of receptor activator of NF-*κ*B ligand by denosumab attenuates vascular calcium deposition in mice. *The American Journal of Pathology*.

[214] Saag KG, Shane E, Boonen S (2007). Teriparatide or alendronate in glucocorticoid-induced osteoporosis. *The New England Journal of Medicine*.

[215] Shin MY, Kwun IS (2013). Phosphate-induced rat vascular smooth muscle cell calcification and the implication of zinc deficiency in a7r5 cell viability. *Preventive Nutrition and Food Science*.

[216] Hiruma Y, Inoue A, Hirose S, Hagiwara H (1997). Angiotensin II stimulates the proliferation of osteoblast-rich populations of cells from rat calvariae. *Biochemical and Biophysical Research Communications*.

[217] Guan XX, Zhou Y, Li JY (2011). Reciprocal roles of angiotensin II and angiotensin II receptors blockade (ARB) in regulating Cbfa1/RANKL via cAMP signaling pathway: possible mechanism for hypertension-related osteoporosis and antagonistic effect of ARB on hypertension-related osteoporosis. *International Journal of Molecular Sciences*.

[218] Armstrong ZB, Boughner DR, Drangova M, Rogers KA (2011). Angiotensin II type 1 receptor blocker inhibits arterial calcification in a pre-clinical model. *Cardiovascular Research*.

